# Status of Iranian schools’ psycho-social environment: cultural adaptation and validation of the Persian version of the W.H.O profile to create Child-Friendly Schools

**DOI:** 10.1186/s12889-022-14260-z

**Published:** 2022-10-04

**Authors:** Amin Ahrari, Fatemeh Salmani, Tayebeh Zeinali, Kimia Izadi, Azam Yousefi, Fatemeh Rahimi, Ensiyeh Norozi

**Affiliations:** 1 Faculty of Health, Department of Health Education and Health Promotion, Social Determinants of Health Research Center, Birjand University of Medical Sciences, Birjand, Iran; 2 Department of Epidemiology and Biostatistics, School of Health, Social Determinants of Health Research Center, Birjand University of Medical Sciences, Birjand, Iran; 3 Infectious Diseases Research Center, Birjand University of Medical Sciences, Birjand, Iran; 4 Department of Health Education and Health Promotion, Yasuj University of Medical Sciences, Yasuj, Iran; 5 Department of Health Education and Health Promotion, Social Determinants of Health Research Center, Birjand University of Medical Science, Birjand, Iran; 6 Department of Health Education and Health Promotion, School of Health, Hormozgan University of Medical Sciences, Bandar abbas, Iran; 7 Department of Public Health, Social Determinants of Health Research Centre, School of Health, Birjand University of Medical Sciences, Birjand, Iran

**Keywords:** Adaptation, Cross-Cultural comparison, Iran, Psycho-social environment, Schools, Validation study

## Abstract

**Background:**

Creating an environment for emotional and social well-being is an important responsibility of Health-Promoting and Child-Friendly Schools. Thus, the present study aimed to assess cultural adaptation and validation of the Persian version of the Psycho-Social Environment (PSE) Profile. The second purpose of this study was to survey the psycho-social environment of schools among a local sample of Iranian school staff.

**Methods:**

This study was conducted in two phases, including cultural adaptation and validation of a culturally adapted scale. The cultural adaptation process followed the procedure suggested by Beaton et al. Then, the culturally adapted scale was administered to a local sample of Iranian school staff including managers (21.9%), teachers (57.4%), support staff (4%), and other school staff (16.7%) in a cross-sectional study. The participants’ mean age was 39.98 ± 8.11 years and they were mostly female (62.8%). The psychometric properties of the culturally adapted version of the questionnaire were tested using a confirmatory factor analysis (*n* = 265), and a test of internal consistency. Finally, the status of schools’ psycho-social environment was assessed using descriptive and analytical statistics.

**Results:**

Confirmatory factor analysis indicated an overall good fit for the 7-factor profile (χ2/df: 1.906, PNFI: 0.62, TLI: 0.78, CFI: 0.79, RMSE: 0.059). The test of internal consistency showed an acceptable reliability (α = 0. 98).

**Conclusions:**

The Persian version of the PSE profile was culturally adapted for use in Iranian schools. Certainly, this culturally adapted version of PSE profile could be useful to determine the school psycho-social environment and to make any changes that can promote a friendly school climate for all participants, and to enhance learning and development.

**Supplementary Information:**

The online version contains supplementary material available at 10.1186/s12889-022-14260-z.

## Background

Nowadays, there is an increasing recognition that schools play an important role in physical, psychological, social, and intellectual development of children and adolescents [1–3]. Based on the growing body of research, there is inextricable association between different aspects of students’ health and school psycho-social environment [2–6]. For example, a literature review showed that the positive school climate is associated with students’ healthy growth, academic achievement, effective violence prevention, and teacher retention [4]. Evidence from another literature review shows that positive school climate is related to positive student development, student learning and can promote cooperative learning, group cohesion, respect, and mutual trust [5]. Pierce [3] also emphasized that school environment has a great impact on student psychological well-being and desired educational outcomes. The Dakar Framework for Action recommends a school climate that not only encourages learning but is friendly, warm, gender-sensitive, safe and healthy. Such schools can enhance the teacher and learner psycho-social and emotional health [2, 7]. World Health Organization (WHO) Information Series on School Health (2003) also emphasizes the importance of a healthy psycho-social environment in schools [2]. Thus, creating an environment for emotional and social well-being is an important responsibility of schools.

There are many ways to measure school climate including school checklists, self-report measurement of climate by students, teachers, administrators, or parents and other observational strategies [8]. There are also different measurement scales for school climate including the California School Climate Survey [9], the Maryland Safe and Supportive Schools Climate [10, 11] and the Psycho-Social Environment (PSE) Profile [2]. But, due to the lack of comprehensive Persian standardized instrument for measuring school climate, there is few studies in Iran about the comprehensive assessment of school psycho-social environment [11, 12].

One of the most comprehensive scales for school climate measurement is the Psycho-Social Environment (PSE) profile developed by WHO to assess healthy psycho-social environment at schools [2]. This profile is developed to help school staff to recognize different aspects of the school environment that affect students’ social and emotional well-being and to create a safe and supportive school environment. According to this profile, the features of the school psycho-social environment are providing a warm, friendly and rewarding learning experiences, promoting active and cooperative learning experiences, facilitating supportive and open communications between school and home life through involving parents, valuing the provision of creative activities, forbidding physical punishment, not tolerating violence and bullying, and finally promoting equal opportunities for boys and girls [2]. Considering the significant effect of a healthy school climate on students’ health, the use of the PSE profile developed by WHO is essential to assess the school climate in Iran. For this reason, the present study is focused on the cultural adaptation and validation of the Persian version of PSE Profile. Also, the other purpose of this study was to survey the psycho-social environment of schools among a local sample of Iranian school staff.

## Methods

This study was conducted in two phases, including cultural adaptation first and then the validation of the culturally adapted scale. The PSE profile contains a series of questions (98 items for Single sex school; 114 items for mixed sex schools) and these questions are all grouped into seven “quality areas” [2]. The name of each quality area and the number of items within each are provided in Table 1. It should be noted that all the schools investigated in this study were single sex. The score for each question ranged from 1 (not at all) to 4 (very much).

**Table 1:** The number of questions in each area of the PSE Profile

Quality Areas	Number of Items in Single sex school	Number of Items in Mixed sex schools
**Providing a friendly, rewarding and supportive atmosphere**	18	24
**Supporting cooperation and active learning**	8	10
**Forbidding physical punishment and violence**	20	21
**Not tolerating bullying and harassment**	18	18
**Valuing the development of creative activities**	10	12
**Connecting school and home life**	13	13
**Promoting equal opportunities and participation**	11	16
**TOTAL**	98	114

### Phase 1: cultural adaptation

The cultural adaptation process followed the steps suggested by Beaton et al. 2000 [13]. These included the initial translation, synthesis of translations, back translation, expert committee review and testing the pre-final version**.**

### Step 1: initial translation

The first step of a cross-cultural adaptation is forward translation. As recommended by Beaton, at least two independent translators should translate the instrument from the source language to the target language. One should have the knowledge about the concepts of the questionnaire being translated, and the other not knowledgeable about the concepts being examined [13]. In this study, the initial translation into Persian was done by two Iranian bilingual health promotion specialists who were familiar with the concepts within the profile and also an Iranian bilingual translator with no background in the concepts being examined in the questionnaire being translated.

### Step 2: synthesis of the translations

The next step of the cross-cultural adaptation is the synthesis of translation results to come up with a single translation [13]. In this study, three forward translators, an independent translator and the main researcher (EN) synthesized the results of the three translations into a single translation based on the original scale and resolved the ambiguities and discrepancies through a consensus.

### Step 3: back translation

The third step of cultural adaptation is back translation, which means translating back from the synthesized translation of target language into the source language. Back-translators should neither be aware nor informed of the concepts being examined, and should be native speakers of the source language (English) as their mother tongue [13]. In this study, the synthesized adapted Persian version was back translated into English by a translator who was native-speaker English and worked independently. He was also neither aware nor was informed of the concepts explored. It is noteworthy that based on the Beaton’s recommendation, at least two independent back translators must be translating the instrument from the target language to the source language [13], but because of some constrains, only one translator for back translation was used in this study.

### Step 4: expert committee review

The fourth step in the process of cultural adaptation is consulting with a panel of experts to evaluate and resolve discrepancies between the source and the final adapted version. The aim of forming and consulting the panel of experts is to develop the pre-final version of the profile for field testing [13]. The panel should make sure that the scale has used the right equivalents for the meaning of words, the target cultural context, concept and experiences of the target culture. In this study, the panel of experts consisted of eight experts – four health educators, one psychometric specialist and three forward translators (except the back translator) – who reviewed all the translations and compared them with the original scale to reach a consensus on discrepancies and produce a pre-final Persian draft of the PSE Profile.

### Step 5: testing the pre final version

The fifth step of adaptation process is the pretesting of the prefinal draft of the instrument in subjects from the target population. As recommended by Beaton, 30–40 subjects need to be tested [13]. Each subject completes the questionnaire, and is interviewed to ensure understandability of the final items and to find out which items are inappropriate or confusing [13]. In this study, the questionnaires are administered directly to 30 subjects (participants ‘mean age was 35.20 ± 7.13 years and they were mostly teachers (63%) followed by managers (31%), and support staff (6%) to ensure whether the target population could understand the adapted version properly. Following the pretest, the final draft of the profile was prepared for a field test in a cross-sectional study.

### Phase 2: validation study

It is highly recommended that after the cultural adaptation process, the psychometric properties of the new culturally adapted instrument be tested and substantiated [13, 14]. For this reason, the culturally adapted PSE Profile was administered to a representative sample of Iranian school staff including managers, teachers, and support staff in a cross-sectional study. Then the psychometric properties of the culturally adapted profile were tested in a confirmatory factor analysis, as well as a test of internal consistency.

### Participants

This study was carried out from April to August 2021 in South Khorasan, a province in the east of Iran. A cluster random sampling was used for data collection. Eleven cities of South Khorasan province were considered as clusters and the number of schools selected from each city was proportional to the number of schools in each city. According to the PSE guideline, the number of subjects asked to fill out the PSE Profile varies across schools. Small schools can invite all school staff to respond. Large schools are better to select a random sample of school staff to save time and effort [2]. Based on the Morgan table, 155 schools were considered to be included in this study, but according to the financial and human constraints, the questionnaires were completed only by 350 subjects from 72 schools. The mean age of participants was 39.98 ± 8.11 years and they were mostly female (62.8%). The majority of participants were teachers (57.4%), followed by managers (21.9%), other school staff (16.7%), and support staff (4%). It is noteworthy that due to the outbreak of COVID-19, the study was performed as an online survey. For this reason, the online version of the PSE profile was developed in Google form. Then, an invitation containing a description of the purpose of study, along with the online hyperlink was sent to all participants in WhatsApp (a highly popular social network application in Iran). Before completing the online PSE profile, an electronic consent form was signed by all participants. The time for responding to the online questionnaire was around 15-min.

### Confirmatory Factor Analysis (CFA)

The underlying factors of the culturally adapted profile was assessed via CFA (using AMOS 24; AMOS development Corp., Crawford, FL, USA). As suggested by Hu and Bentler (1999), the following indices were used to assess the goodness-of-fit of the data: Comparative Fit Index (CFI) with a cut-off value of CFI ≥ 0.90, Tucker– Lewis Index (TLI) with a cut-off value of TLI ≥ 0.90, the normed χ2 with a cut-off value of normed χ2/df < 5, Root Mean Squared Error of Approximation (RMSEA) with a cut-off value of RMSEA ≤ 0.08, and Parsimonious Normed Fit Index (PNFI) with a cut-off value of PNFI ≥ 0.5 [15].

### Reliability assessment

The reliability of the profile was assessed using Cronbach’s Alpha coefficient. An alpha coefficient greater than 0.70 was considered to represent the internal consistency of the scale [16].

### Statistical analysis

The data were analyzed using SPSS 24 (SPSS Inc. released 2009. PASW Statistics for Windows, Chicago: SPSS Inc.). The quantitative variables were described by mean and standard deviation (SD) and the qualitative variables were described by frequency and percentage. Pearson’s correlation coefficient was used to show the linear relationship between each quality area of the profile. The significant level was set at *P* < 0.05.

## Results

### Phase 1: cultural adaptation

In the process of cultural adaptation, 7 items were removed from the PSE profile. Indeed based on the view of expert committee, 3 items from quality area 4 (Not tolerating bullying, harassment and discrimination) were not in line with the ethical and legal considerations in Iran’s educational system and should be removed from the list of items. However, because of the PSE guideline that emphasized not to change or delete any part of the profile, the panel of experts decided to retain these items. In the next step and before testing the pre-final draft with the research participants, we needed to gain an ethical approval from the department of education. Unfortunately, the security office of the department of education only allowed us to use the PSE profile once 3 items were removed from quality area 4 (Not tolerating bullying, harassment and discrimination) and 4 items from quality area 7 (Promoting equal opportunities and participation in decision-making). As commented by the Ethics Committee of the Department of Education, these items were not in line with the ethical and legal considerations in Iran’s educational system Once these were removed, the questionnaire was administered directly to 30 participants to ensure whether the target group could adequately understand the adapted version or not. Following the pretest, the final draft of questionnaire (91 items) was prepared for the field test in a cross-sectional study.

### Phase 2: validation study

The quality of the factors of the 91-item profile was confirmed through a CFA on the 265. As noted above, a total number of 350 participants completed the survey form. However, 85 participants were excluded from the final analysis as they had missed more than 2 items on the profile. As shown in Table 2, the goodness of fit indices indicated an overall good fit for the construct of the 7-factor profile.

**Table 2:** Goodness of fit indices of the PSE profile (*n* = 265)

Criteria	χ2/df	PNFI	TLI	CFI	RMSE
value	1.906	0.62	0.78	0.79	0.059
threshold	< 5	> 0.5	> 90	> 90	< 0.08
Decision	Accepted	Accepted	Accepted	Accepted	Accepted

**Fig. 1:**
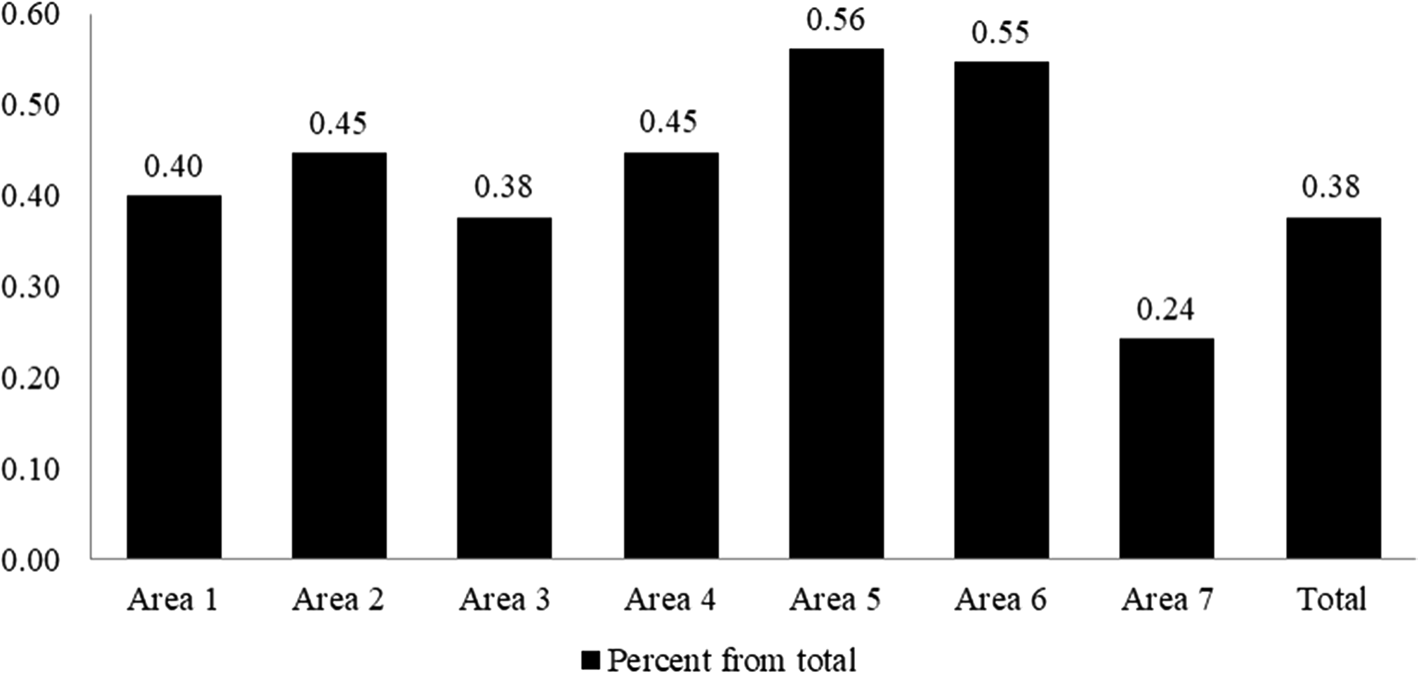
The desirable percent of each quality area of PSE profile

Figure 1 shows the desirable percent of each quality area of PSE profile‍‍‍‍. For example, the most desirable score of the first quality area with the maximum score of the questionnaire is 72. On average, only 40% of the desired conditions in this area have been met in this study (mean ± SD in area 1: 28.76 ± 10.06). In the second quality area, the average score is 14.28 ± 4.25. This explains 45% of the desired condition. The third quality area only met 38% of the proper condition with an average score of 30.04 ± 9.33. The fourth, fifth and sixth area met 45, 56 and 55% of the desired condition, respectively. The minimum of the desired condition was obtained in the seventh area as 38%.

The internal consistency of the profile and its 7 quality areas was confirmed by calculating the Cronbach’s alpha coefficient values (Table 3).

**Table 3:** Number of Items, Total score and reliability scores of questionnaire domains (*n* = 265)

	Number of Items	Total score		Cronbach’s alpha
Quality Area 1	18	72	Providing a friendly, rewarding and supportive atmosphere	0.94
Quality Area 2	8	32	Supporting cooperation and active learning	0.80
Quality Area 3	20	80	Forbidding physical punishment and violence	0.92
Quality Area 4	15	52	Not tolerating bullying and harassment	0.94
Quality Area 5	10	36	Valuing the development of creative activities	0.93
Quality Area 6	13	40	Connecting school and home life through involving parents	0.93
Quality Area 7	7	60	Promoting equal opportunities and participation in decision-making	0.83
Total	91	372		0.98

As shown in Table 4, there are high and significant linear correlations between the 7 quality areas of the PSE profile and the overall score.

**Table 4:** Correlations between the 7 quality areas of the PSE profile and the overall score

	Quality Area 1	Quality Area 2	Quality Area 3	Quality Area 4	Quality Area 5	Quality Area 6	Total
Quality Area 1	.764^**^	.745^**^	.740^**^	.614^**^	.715^**^	.603^**^	.713^**^
Quality Area 2		.685^**^	.719^**^	.622^**^	.692^**^	.620^**^	.757^**^
Quality Area 3			.823^**^	.570^**^	.701^**^	.620^**^	.818^**^
Quality Area 4				.640^**^	.692^**^	.666^**^	.886^**^
Quality Area 5					.689^**^	.626^**^	.793^**^
Quality Area 6						.729^**^	.872^**^
Quality Area 7							.784^**^

^**^ *p* < 0.001

## Discussion

WHO developed a psycho-social environment profile to help school staff, parents, and students to enhance school quality by creating a healthy psycho-social climate in schools [2]. The present study aimed to assess the validity and reliability of the cultural adapted Persian version of the PSE Profile. In general, the present findings showed that the Persian version of the PSE Profile is valid and reliable. Also, the present study showed that Iranian schools got relatively low to moderate scores in all seven quality areas. The lowest score was for quality area 7 and the highest for quality area 5. In below, details on each quality area of the profile were discussed.

### Quality Area 1. Providing a friendly, rewarding and supportive atmosphere

The ‘friendly, rewarding and supportive climate’ of a school is one of the most important features of a good school [2]. The role of school climate in promoting children’s mental health has been emphasized in many studies and international policy documents [2, 17]. A positive school climate was found to be associated with better psychological well-being [18]. It was also emphasized that a positive school climate plays an important role in effective risk prevention and health promotion interventions [4]. The present findings showed that all items of quality area 1 are culturally adapted with Iranian culture. Our results also showed that Iranian schools got a relatively moderate score in this quality area. Indeed, based on the view of most participants, their schools did not provide a friendly, rewarding, and supportive climate. The school staff, especially teachers could be the role models for promoting the respect and positive relations at school and to establish a strong sense of belonging to the school among all those who work and study there [19]. These results could be used by Iranian school staff to assess more carefully the conditions in their own school, to make any changes that would help to improve the school capacity to be supportive and caring, and to establish a culture of inclusion and respect that welcomes all those who work there.

### Quality area 2. Supporting cooperation and active learning

Promoting cooperative and active learning is central to the creation of a more health promoting climate [2]. It is an important way of empowering students to take more responsibility for their own learning. It can also improve relations between students from different social and ethnic groups [2, 20]. The more positive relationships that result from cooperative learning tend to increase the sense of responsibility to the group and school, increase willingness to take on difficult tasks, increase commitment to each other’s academic success, and increases academic productivity [20]. The present findings showed that all items of this quality area were culturally adapted with Iranian culture and none were removed in either phase of research. Also, our results showed that Iranian schools got a relatively moderate score in this quality area and most participants stated their schools did not have the right strategies to support cooperation and active learning. As noted above, active and/or cooperative learning has many benefits. Thus, it is really important that all teachers use active and cooperative learning techniques to empower students to take more responsibility of their own learning. These results could be used by school administrators and teachers to make policy (or documentation) on how to promote co-operative learning in their own school, to make any changes that would assist students in learning to treat themselves, others, and their community with compassion, and to build a sense of togetherness among the students.

### Quality area 3. Forbidding physical punishment and violence

Prohibiting physical punishment and violence is another important aspect of school psycho-social environment. There is growing evidence that physical punishment is associated with high rates of mental health problems including drug abuse later on in adulthood [2]. Our results showed that all items of this quality area are culturally adapted with Iranian culture. Our results also showed that Iranian schools got a low score in this quality area. Indeed, as the present participants viewed it, most Iranian schools do not have a formal policy to forbid physical punishment and violence. The results of a study entitled as “Moral conflicts in Iranian secondary schools” also showed that physical punishment is a common and acceptable disciplinary procedure in Iranian schools [21]. In this study, moral conflicts in Iranian secondary schools were examined based on essays and interviews with students and teachers. A total of 310 stories were coded, using deductive and inductive content analysis. As perceived by students, the staff’s unfair and aggressive punishments were the most controversial issue [20]. Based on a national report about Iran published by the End Corporal Punishment (critical initiative of the Global Partnership to End Violence against Children), the clear policy against corporal punishment in Iranian schools should be confirmed through a law reform which clearly prohibits corporal punishment in all education settings, public and private, at all levels [22].

### Quality area 4. Not tolerating bullying, harassment and discrimination

Bullying, harassment and discrimination are important reasons that make attending school a deeply unpleasant experience, and, if continued, can have a destructive effect on student well-being [2, 23]. Indeed, bullying in schools creates negative experiences such as distress, fear, anxiety, anger, and helplessness [24]. As commented by the Ethics Committee of the Department of Education, 3 items of this quality area were removed from the final questionnaire. Although, sexual harassment may occur among Iranian female students and teachers, but 3 related items of this quality area (“Female students are not subjected to sexual harassment at school”, “Female teachers are not subjected to sexual harassment at school”, and “The school has a policy on how to deal with the victims of sexual harassment”) are not in line with ethical considerations in Iran’ educational system. So, these items were removed from the list of items. Other items related to this quality area were culturally adapted with Iranian culture. Our results showed that Iranian schools got a relatively moderate score in this quality area and most participants stated their schools did not have any formal policy about not tolerating bullying, harassment and discrimination. Iran’s educational system is expected to make a formal policy on how to deal with bullying, harassment and discrimination in Iranian schools. Our findings could be also used by school administrators and teachers to create a code of conduct about how the school expects students and teachers to behave, and implement awareness raising strategies to ensure all staff and students know their rights and responsibilities to cater for a safe environment.

### Quality area 5. Valuing the development of creative activity

Another important feature of a health-promoting school is the availability of opportunities for students to participate in creative and recreational activities. It is necessary for children’s health and well-being to have enough fun to promote creativity and imagination. It also helps them to build confidence and realize their potentials [2, 25]. Our results showed that all items of this quality area are culturally adapted with Iranian culture. Also, our results showed that Iranian schools got a moderate score in this quality area. Indeed, as the participants commented, most Iranian schools value the development of creative activity. However, it seems necessary for teachers to understand the significance of creativity for student learning and the various measures that teachers can take during the educational course at school to foster student’s creative expression.

### Quality area 6. Connecting school and home life by involving parents

Parents’ engagement in school activities and decisions is a key element of a health-promoting school [2]. Findings of many studies show that parent engagement in schools is closely related to higher academic achievement [26], enhanced social functioning [27], and fewer unhealthy behaviors, such as smoking behavior [28]. Our results showed that all items of this quality area are culturally adapted with Iranian culture and none of the items were removed at any phase of the work. Also, our results showed that Iranian schools got a moderate score in this quality area and almost most participants stated that their schools have regular opportunities to connect school and home life through involving parents. Schools can encourage parental involvement by treating parents as their partners. This partnership needs a commitment between teachers and parents. For this reason, parents must be committed to prioritizing their child’s educational goals, and teachers committed to listening and providing a space for collaboration with parents.

### Quality area 7. Promoting equal opportunities and participation in decision-making

Finally, another important feature of a health-promoting school is to help students to actively engage in the decision-making process together with the staff and parents. A health-promoting and child-friendly school gives students the opportunity to be informed about the issues that affect them and help them to acquire the confidence they need to stand up for their rights [2]. Our results showed that 4 items of this quality area (“The materials and resources used by students are free from pejorative ethnic stereotypes”, “The materials and resources used by students are free from religious stereotypes.”, “The materials and resources used by students are free from gender stereotypes.”, and “Students take part in activities that help them to recognize, understand and value differences between them (e.g., cultural, religious and social.”) are not in line with ethical considerations in Iran’s educational system. Thus, these items were removed from the list of items. Other items of this quality area were culturally adapted to Iranian culture. The present findings also showed that Iranian schools got a very low score in this quality area. Indeed, as the participants commented, most Iranian schools fail to promote equal opportunities and engagement in decision-making. Engagement in decision-making mechanisms at school brings students many positive outcomes such as developing leadership skills, increasing academic achievement, increasing self-confidence, and improving student life skills such as taking responsibility, communicating effectively, problem solving and so on [29].

## Conclusion

Based on the present findings, the Persian draft of the PSE profile is culturally adapted for use among Iranian schools. Certainly, this cultural adapted version of the PSE Profile could be useful for determining school psycho-social environment status and to make any changes that would promote the school friendly climate for all relevant participants, and to enhance learning and development.

Also, the present findings showed that Iranian schools got relatively low to moderate scores in all seven quality areas. Our results could inform school administration, teachers, students and parents of the psycho- social qualities of their school environment. They could be requested to discuss the results and provide advice for headmasters about recognizing positive achievements and making improvements as needed. Also, the school staff may need to be encouraged and further trained to take the best advantage of the PSE profile.

### Limitations

It is important to note that there are several limitations in this study. Firstly, the present participants were solely the school staff of south Khorasan (an east Iranian province); therefore, a cross-validation of the PSE profile with a larger and more nationally representative sample of school staff from the other provinces of Iran is necessary before making any claims about the generalizability of the questionnaire. Secondly, the recruitment of respondents was from among a group of school staff who had previously expressed their desire to participate in this study. This suggests that probably there was some selection bias including the school staff with a high interest in the school activities. Finally, because the PSE Profile is designed to be completed by school staff, students were not involved in any phase of this study. Yet, it is recommended to involve students in discussions of items in the Profile that relate to the experience and perceptions of students. Indeed, the PSE Profile can have large benefits if it is completed by students to assess the psycho-social environment of their own school and to make any change that would assist in promoting a friendly climate to them.

## Supplementary Information

**Additional file 1. **W.H.O Psycho-Social Environment (PSE) Profile.

## Data Availability

All data generated or analyzed during this study are included in this published article.
