# Supplementary material for: Status of Iranian schools’ psycho-social environment: cultural adaptation and validation of the Persian version of the W.H.O profile to create Child-Friendly Schools
Source: BMC Public Health. 2022 Oct 4;22:1856. doi: 10.1186/s12889-022-14260-z (PMC9531853; doi:10.1186/s12889-022-14260-z)
Supplement: Supplementary file 1 — Additional file 1. W.H.O Psycho-Social Environment (PSE) Profile. [file 12889_2022_14260_MOESM1_ESM.pdf]

THE PSYCHO-SOCIAL (PSE) PROFILE QUESTIONNAIRE

A positive school environment can promote healthy social and emotional development during the early years of life. The items and information contained in this PSE Profile were derived in large part from a systematic review of evidence from more than 650 research articles in the international literature and the original Profile that was reviewed by schools in 20 countries worldwide. You are invited to fill out the PSE Profile by answering questions about this school that are grouped into **seven "quality areas":**

1. Providing a friendly, rewarding and supportive atmosphere
2. Supporting cooperation and active learning
3. Forbidding physical punishment and violence
4. Not tolerating bullying, harassment and discrimination
5. Valuing the development of creative activities
6. Connecting school and home life through involving parents
7. Promoting equal opportunities and participation in decision-making.

Please begin by providing the following information about yourself:

What is your role at the school? ❑ Manager ❑ Teacher ❑ Support staff ❑ other

What is your sex? ❑ Male ❑ Female

Instructions

Please answer each question by circling ONE of the four possible answers. Choose the answer that you feel best describes your school. Do not spend a lot of time thinking about the answer — usually your first reaction is the best. There are no right or wrong answers; we just want to know what you think about your school. Please make sure that you have answered all the questions.

Quality Area 1. Providing a friendly, rewarding and supportive atmosphere					
	**Item**	**Not at all**	**A little**	**Quite a lot**	**Very much**
**1**	**The school is friendly and welcoming to visitors**				
**2**	**Students are encouraged to welcome and assist newcomers to the school**				
**3**	**The school has a policy on how to integrate new students into the school**				
**4**	**The school is seen as an appealing place to work by those who work there.**				
**5**	**Staff encourages the students to care for each other.**				
**6**	**Teachers support students who are in distress.**				
**7**	**There is a trusted person who the students know they can approach if they have a problem or need confidential advice.**				
**8**	**The school holds regular events where the achievements of students are publicly recognized and applauded.**				
**9**	**Feedback about a student's work is accompanied by positive comments about achievements and suggestions for improvement.**				
**10**	**Teachers are confident that they will receive help and support from other staff when they need it.**				
**11**	**Teachers are treated in ways that help them to develop and maintain their self-confidence as educators.**				
**12**	**Staff behaves in a purposeful and orderly manner.**				
**13**	**Students are confident that they will get help and support when they need it.**				
**14**	**Staffs have a strong sense of belonging to the school.**				
**15**	**Students have a strong sense of belonging to the school.**				
**16**	**Parents are interested in and supportive of the school and its governance.**				
**17**	**Staff is concerned about what happens to each other.**				
**18**	**Students are concerned about what happens to each other.**				

Quality Area 2. Supporting cooperation and active learning.					
	**Item**	**Not at all**	**A little**	**Quite a lot**	**Very much**
**1**	**There is a school policy (or documentation) on how to promote co-operative learning (e.g., using teaching methods that encourage the students to participate in class).**				
**2**	**Students spend time working together to solve problems.**				
**3**	**Students are encouraged to ask questions in the classroom.**				
**4**	**Teachers organize students for group activities so that they can work together.**				
**5**	**Teachers are seen to be co-operating with each other.**				
**6**	**The school discourages announcing the order of students in each class, based on their academic performance.**				
**7**	**Students work on projects for and with their local community.**				
**8**	**The students' work is regularly put on display.**				

Quality Area 3. Forbidding physical punishment and violence.					
	**Item**	**Not at all**	**A little**	**Quite a lot**	**Very much**
**1**	**The school has a policy prohibiting physical punishment as an acceptable disciplinary procedure.**				
**2**	**The school has a policy promoting non-physical punishment as an acceptable disciplinary procedure.**				
**3**	**Teachers avoid using physical punishment to discipline children.**				
**4**	**Teachers are supported in the use of non-aggressive styles of discipline.**				
**5**	**The school keeps records of disruptive incidents that occur during the day.**				
**6**	**The school has policies and procedures to help teachers deal fairly and consistently with aggression and violence.**				
**7**	**The school has policies (short and long term) about how to deal with the consequences of violent incidents.**				
**8**	**School policies for dealing with violence and aggression are enforced.**				
**9**	**Teachers have the opportunity to gain new knowledge and skills that help them to maintain a safe and secure school.**				
**10**	**Support is available for teachers who have been involved in violent or stressful incidents.**				
**11**	**The school has procedures to deal with students/school staff who have witnessed violence.**				
**12**	**The school discipline rules are clear to everyone.**				
**13**	**The school discipline rules are practical.**				
**14**	**Discipline is maintained well at the school.**				
**15**	**Students feel safe in school.**				
**16**	**Teachers feel safe in school.**				
**17**	**Those in charge are seen as firm, fair and consistent.**				
**18**	**There is a procedure that allows all students to voice concerns about inappropriate or abusive behavior.**				
**19**	**There is a procedure that allows parents to voice concerns about inappropriate or abusive behavior.**				

**20**	**The school recognizes good behavior practiced by students.**				

**Quality Area 4. Not tolerating bullying, harassment and discrimination**					
	**Item**	**Not at all**	**A little**	**Quite a lot**	**Very much**
**1**	**The school has a publicized policy that bullying will not be tolerated.**				
**2**	**The school has a publicised policy that harassment will not be tolerated.**				
**3**	**The school has publicized procedures on how staff should intervene if bullying arises.**				
**4**	**The school policies for dealing with bullying are enforced.**				
**5**	**The students know that the school disapproves of bullying in school, outside school and while travelling to and from school.**				
**6**	**The students know that they can seek help from named staff members if they are bullied.**				
**7**	**The school has a policy on how to deal with the victims of bullying.**				
**8**	**Female students are not subjected to sexual harassment at school.**				
**9**	**Female teachers are not subjected to sexual harassment at school.**				
**10**	**The school has a policy on how to deal with the victims of sexual harassment.**				
**11**	**There is a code of conduct about how the school expects students to behave.**				
**12**	**There a code of conduct about acceptable behaviour between staff and students.**				
**13**	**The code of conduct is regularly updated.**				
**14**	**All policies and codes of conduct are displayed in the school for everyone to read.**				
**15**	**Staff have a policy about the best way to include ‘loners’ and those who are recognized as 'different’ in school activities.**				
**16**	**The staff take active steps to prevent the exclusion of students by their peers.**				
**17**	**The school records and monitors injuries reported by students and staff.**				
**18**	**The school has student advisors who have been trained to mediate when conflict occurs.**				

Quality Area 5. Valuing the development of creative activities.					
	Item	Not at all	A little	Quite a lot	Very much
1	There are regular times available for recreation and play throughout the school day.				
2	The play and recreation periods are supervised by responsible adults.				
3	There are sufficient supervisors to monitor activities in every part of the play area.				
4	There are sufficient supervisors to monitor activities in every part of the play area.				
5	There is the opportunity for students to create their own imaginative games without involving adults.				
6	All students have opportunities to experience creative learning experiences that are free from the stress of competition and examinations, e.g. music, art, drama.				
7	All students have opportunities to experience creative learning experiences that involve reasonable, constructive competition.				
8	All students have opportunities to experience creative learning experiences that provide rewards for effort as well as achievement.				
9	All students are provided with opportunities to engage in physical activity as a recreational choice.				
10	There is a programme of activities outside the school hours that students can join.				

Quality Area 6. Connecting school and home life through involving parents					
	Item	Not at all	A little	Quite a lot	Very much
1	Parents are informed about policies and codes of conduct in the school.				
2	Parents are encouraged and assisted by the school to help their children consolidate their learning at home.				
3	Parents know that the school should be told about any major changes in the child's home life so that help can be provided, if needed.				
4	The school invites parents to discuss the child's work with the teachers.				
5	Parents feel welcome at the school.				
6	Parents have the opportunity to discuss the school's policies and codes of conduct and to contribute to decision-making by the school.				
7	Parents know that the school actively promotes cooperation, inside and outside the classroom.				
8	Parents feel able to go to the school to ask questions or discuss worries they have about their child.				
9	There are regular opportunities for parents to inform the teacher and other appropriate authorities about what is happening at home and in the community.				
10	There are opportunities for parents to be involved in activities linked to the school life and work, e.g., outings, fund-raising.				
11	Parents are involved in discussion and decisions about what students are taught at school.				
12	Parents are involved in discussion and decisions about how students are taught (i.e., teaching methods).				
13	The school regularly communicates news to parents about the school and its activities.				

Quality Area 7. Promoting equal opportunities and participation in decision-making					
	**Item**	**Not at all**	**A little**	**Quite a lot**	**Very much**
**1**	**Students have the opportunity to speak, and be listened to, in class**				
**2**	**There is a procedure that enables all students to openly express their feelings and thoughts about school work and school life.**				
**3**	**The school actively involves students in decisions about how the school is organized.**				
**4**	**The materials and resources used by students are free from pejorative ethnic stereotypes.**				
**5**	**The materials and resources used by students are free from religious stereotypes.**				
**6**	**The materials and resources used by students are free from gender stereotypes.**				
**7**	**Students take part in activities that help them to recognize, understand and value differences between them (e.g., cultural, religious and social).**				
**8**	**Students who are 'different' in any way are treated with respect and equality.**				
**9**	**No students in the school are excluded from the possibility of being successful.**				
**10**	**Students take part in deciding the rules of the school.**				
**WHERE APPROPRIATE:**					
**11**	**Students who are working in their second language have opportunities during the school day to speak in their first language.**				
**12**	**Girls and boys are treated as equals.**				
**13**	**Girls and boys have the same opportunities to reach their potential.**				
